# Lactic alkalosis in intensive care: a red flag?

**DOI:** 10.1186/s13054-023-04464-z

**Published:** 2023-05-12

**Authors:** Auguste Dargent, Florent Wallet, Arnaud Friggeri, Julien Bohe, Charles-Hervé Vacheron

**Affiliations:** 1 Centre Hospitalier Lyon Sud, Département d’Anesthésie Réanimation Médecine Intensive, Hospices Civils de Lyon, 165 Chemin du Grand Revoyet, 69310 Pierre-Bénite, Lyon, France; 2 APCSe Aggressions Pulmonaires et Circulatoires dans le Sepsis, UPSP 2016.A101, VetAgro Sup, 69280 Marcy l’Étoile, France; 3 RESHAPE Research on Healthcare Performance, U1290, Université Claude Bernard Lyon 1, Lyon, France; 4 PHE3ID, Centre International de Recherche en Infectiologie, Institut National de la Santé et de la Recherche Médicale U1111, CNRS Unité Mixte de Recherche 5308, École Nationale Supérieure de Lyon, Université Claude Bernard Lyon 1, Lyon, France; 5 Université Lyon 1, Lyon, France

Dear editor,

Alkalosis is the most common acid–base disturbance that develops in critically ill patients [1]. Indeed, the study by Mæhle et al. based on more than 100,000 samples showed that the median base excess (BE) increases steadily during the stay of critically ill patients, due to multiple factors such as diuretics and hypoprotidemia [1]. Despite these findings, alkalosis is still relatively neglected in the field of intensive care research. As an illustrative example, a PubMed search performed on 04/10/2023 showed 10 time less results for [alkalosis AND intensive care] as compared with [acidosis AND intensive care]. In our intensive care unit (ICU), we regularly observe patients with both alkalosis and mild hyperlactatemia, without any obvious cause for hyperlactatemia. We therefore requested through electronic health record (IntelliSpace Critical Care and Anesthesia, Philips^®^) all arterial blood gases associated with lactate measurement performed in ourICU (*Centre Hospitalier Lyon Sud, Hospices Civils de Lyon*), between the January 1, 2014, and the October 31, 2022. The analysis was performed with the appropriate regulatory agreement (CNIL N°2,228,340). After restricting our analysis to pH values of 7.45 or higher, 57,393 samples were identified with the following distribution: 46% (26,578) with pH within 7.45–7.47, 39% (22,223) within 7.48–7.51, 11% (6,023) within 7.52–7.54, 1,718 (3%) within 7.55–7.57, 1% (579) within 7.58–7.60, and less than 1% (272) over 7.6. The median associated lactate measurement was 1.4 IQR [1.0–1.9] mmol/l. After categorizing the pH values along pre-defined cut points, we observed that lactate measurement increased along with pH values (Fig. 1, panel A). Using linear regression, we showed that the ascension of ^1^/_10_ of pH was associated with an increase of 0.33 mmol/l of lactate measurement (*p* < 0.001). The 90th percentile of lactate measurement in the different categories of pH was constantly increasing (as illustrated in Fig. 1 by a red dot) at 2.4 mmol/l, 2.5 mmol/l, 2.7 mmol/l, 2.9 mmol/l, 3.3 mmol/l, and 3.5 mmol/l. Additionally, we used BE to separate metabolic alkalosis (pH > 7.45 and BE > 3 mmol/L, *N* = 29,486 (51%)) from respiratory and mixed alkalosis (pH > 7.45 and BE ≤ 3 mmol/l, *N* = 29,907 (49%)). The same association between serum lactate and pH was present in both categories (Fig. 1, panel B).

**Fig. 1:**
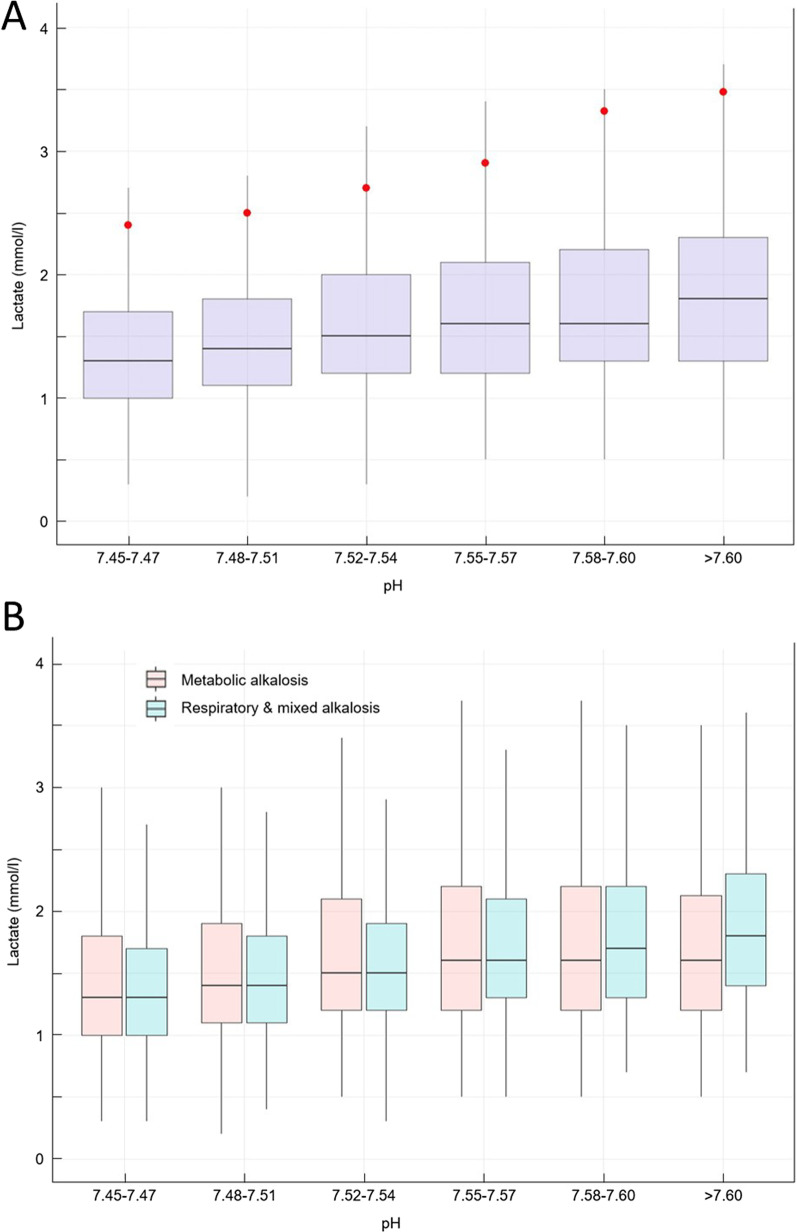
Association between lactate measurement and alkalosis. Panel **A**: Box plot of lactate measurement as a function of the associated pH category. Red dots show the 90th percentile of lactate measurement in each category. Panel **B**: Box plot of lactate measurement according to the category of alkalosis: metabolic (orange), or respiratory

An association between alkalosis and mild hyperlactatemia has been described before in numerous case reports of extreme alkalosis, of both respiratory [2] and metabolic [3] origin. Bersin et al. [3] introduced the concept of “primary lactic alkalosis” in a study describing a small case series along with an experimental demonstration of the pH-induced hyperlactatemia in a swine model of metabolic alkalosis. Several mechanisms are proposed to explain its pathophysiology: First, alkalosis stimulates glycolysis by increasing the activity of phosphofructokinase, which is sensitive to both pH itself and to alkalosis-induced sympathetic hyperactivation; second, alkalosis-induced left shift in the hemoglobin dissociation curve decreases oxygen tissue extraction, which may explain the decrease in oxygen consumption observed in the seminal study by Bersin [3]. During exercise in man, experimental studies observed the same positive correlation between pH and lactate output, which was interpreted as a protective mechanism to regulate cellular acid production and protect muscle pH [4]. In a cohort of patients with psychogenic respiratory alkalosis, hyperlactatemia was present in 30% of patients and was mainly correlated to pH and PaCo_2_ without any relation to adverse events [4], suggesting that “lactic alkalosis” does not carry the same prognostic value as lactic acidosis. In a retrospective cohort study of critically ill patients, alkalosis was not associated with increased mortality but was significantly associated with prolonged ICU stay [5], although lactate was not available, preventing the exploration of its prognosis value in this particular context.

Overall, very little is known about lactic alkalosis in intensive care, and our findings encourage us to explore further the relationship between alkalosis, lactate and outcome in critically ill patients. Nevertheless, it could be useful for the clinician to know that mild, isolated hyperlactatemia in a patient with alkalosis should be interpreted with caution and is not necessarily a “red flag” that should lead to inappropriate therapeutic interventions.

## Data Availability

The datasets used and/or analyzed during the current study are available from the corresponding author on reasonable request.
